# A Mixed Methods Evaluation of Sharing Air Pollution Results with Study Participants via Report-Back Communication

**DOI:** 10.3390/ijerph16214183

**Published:** 2019-10-29

**Authors:** Kathryn S. Tomsho, Claire Schollaert, Temana Aguilar, Roseann Bongiovanni, Marty Alvarez, Madeleine K. Scammell, Gary Adamkiewicz

**Affiliations:** 1 Department of Environmental Health, Harvard T.H. Chan School of Public Health, Boston, MA 02115, USA; malvarez@hsph.harvard.edu (M.A.); gadamkie@hsph.harvard.edu (G.A.); 2 Department of Environmental Health, Boston University School of Public Health, Boston, MA 02118, USA; cscholla@bu.edu (C.S.); aguilart@bu.edu (T.A.); mls@bu.edu (M.K.S.); 3 GreenRoots, Inc., 227 Marginal St., Suite 1, Chelsea, MA 02150, USA; roseannb@greenrootschelsea.org

**Keywords:** data report-back, exposure assessment, mixed-methods evaluation, data communication, community engagement, environmental health, indoor air pollution

## Abstract

We implemented a concurrent triangulation mixed-methods evaluation of an air pollution data report-back to study participants in Chelsea, Massachusetts. We aimed to determine whether the report-back was effective in the following three ways: engagement, understandability, and actionability for the participants. We also evaluated participants’ valuation of the report-back information and process. The evaluation involved both qualitative components, such as ethnographic observation, and quantitative components, such as closed-ended questionnaires and demographic data. The participants who engaged in the report-back process were significantly different from those who did not engage both in terms of their demographics, and in their indoor air pollutant concentrations. Participant understanding generally corresponded with the intended meaning of the research team, suggesting successful data communication. Additionally, many of the participants reported that they were inspired to take action in order to reduce their indoor air pollutant exposure as a result of the report-back process and information provided. These results identify areas of improvement for engagement, particularly regarding populations that may have higher exposures. This work outlines a framework with which to contextualize and evaluate the success of engagement with report-back efforts. Such evaluations can allow research teams to assess whether they are providing information that is equitably useful and actionable for all participants.

## 1. Introduction

Data report-back to participants in environmental health studies is increasingly being incorporated into exposure assessment research [1,2,3,4,5]. The field of environmental health is progressively emphasizing the importance of returning research results to participants [1,2,3,4,5]. Report-back efforts can vary in the types of data presented from individual biomonitoring data (e.g., blood and urine concentrations of chemicals) to broader range exposure data (e.g. pollutants in household dust) [1,6,7,8]. The impetus for providing these data to participants is based in both ethical data ownership considerations, as well as in the practical utility of the data to prompt individual and/or collective action [9]. However, few established tools or methodologies exist to evaluate the effectiveness of data report-back efforts. The work to date has been evaluated primarily through qualitative interviews with research participants who received data back [1,9,10,11,12]. Although qualitative data alone provides insight into the potential impact of data report-back, triangulating qualitative data with quantitative data can bolster the validity of findings by combining the strengths of each approach [13].

The development of a framework identifying the markers of “success” in a report-back effort provides a guide for researchers to evaluate the accessibility and efficacy of their communication materials. Doing so can identify components that may need improvement, and subsequently allow the research team to ensure equitable accessibility to environmental health information. Evaluation is necessary for the development of best practices for the report-back of data to participants. Rigorous evaluation of data report-back efforts can provide insight into the components of the process that were effective, as well as those that may benefit from improvement in the future. Identifying what styles of communication were successful and who engaged in the data report-back effort is a first step in reducing environmental exposures and health disparities. Although the provision of information is not necessarily sufficient for behavioral change or action, it can empower participants to identify potential pollutants within and outside their homes, as well as strategies to mitigate them.

The Handbook for Reporting Results in Biomonitoring and Personal Exposure Studies suggests that the evaluation of report-back efforts is a new field, and that characterizing participant interpretations and perceptions of the results is crucial for improving the accessibility and utility of report-back approaches [14]. The handbook suggests that community meetings may appropriately be evaluated via surveys or questionnaires, and describes an approach that includes evaluating participant experiences and perceptions [14]. We build upon that approach by attempting to capture participant experience and perceptions via qualitative components of our own evaluation framework. Additionally, we identify markers of report-back utility via information gained by participants, and identify indicators of their motivation to act by pre- and post-community meeting questionnaires. As interest in report-back to participants grows in the field of environmental health, it is important to characterize the efficacy of the information provided to improve participant understanding of the data presented. This work is an attempt to characterize our ability to improve participant understanding of their personal data, and their subsequent motivation to reduce their own personal indoor air pollutant exposures.

This evaluation effort assessed the data-report back effort for 72 participants in the Home-based Observation and Monitoring Exposure (HOME) Study in Chelsea, Massachusetts. Outdoor and in-home air quality data were collected across Chelsea, and individual and community results were reported back to participants. The results were communicated via report-back packets individually tailored and mailed to each home, and at an in-person community meeting [15].

### Evaluation Framework

**Figure 1:**
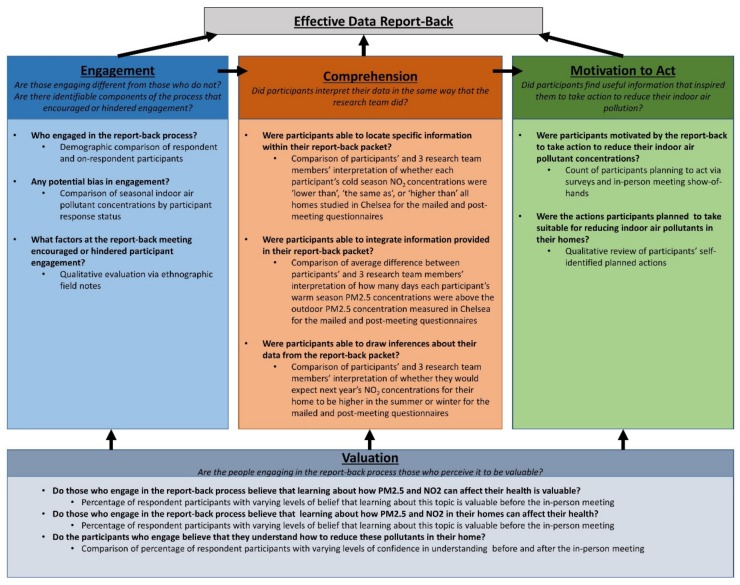
Logic model of effective report-back components.

The data report-back evaluation implemented a concurrent triangulation mixed methods approach in which both qualitative and quantitative information were gathered simultaneously [16]. The overall goals were to characterize the engagement, valuation, comprehension, and motivation of the participants, to act with respect to the data report-back information. Each of these components was considered an important characteristic of a successful data report-back campaign by the research team. Engagement in the report-back process was theorized as a necessary step for the comprehension of the material. Comprehension of the report-back materials was considered important for motivation to action. Valuing the report-back process, broadly, was regarded as a characteristic with the potential to influence participants’ engagement, comprehension, and motivation to act. These proposed pillars of the report-back are outlined in Figure 1.

## 2. Materials and Methods 

### 2.1. Process

The HOME Study, a project within the Center for Research on Environmental and Social Stressors in Housing across the Life Course (CRESSH), measured outdoor PM_2.5_ (particulate matter particles smaller than or at 2.5 micrometers) and nitrogen dioxide (NO_2_) both inside and outside participants’ homes in the City of Chelsea. The HOME study participants hosted an Environmental Multi-pollutant Monitoring Assembly (EMMA) device in their homes for two weeks, one during a cold season and one during a warm season, which recorded the in-home concentrations of PM_2.5_ and NO_2_, among other variables [17]. Upon the completion of field data collection, the pollutant data were synthesized into 24-h averages for each week of monitoring for each participant, for both their PM_2.5_ and NO_2_ concentrations. The research team included Harvard and Boston University Schools of Public Health faculty, students, and staff, and members of GreenRoots staff (a Chelsea-based environmental justice organization). Data report-back packets were mailed to each participating household [15]. 

The evaluation was comprised of multiple components, namely: a questionnaire mailed with the report-back packets, a sign-in table and qualitative note taking at the in-person community meeting, a post-meeting questionnaire, and an optional voicemail line for feedback. The report-back packet contained information presented in both graphical and prose form, on the following: methods of measurement of PM_2.5_ and NO_2_ in their home, their individual home’s 24-h averaged data for both pollutants, HOME Study participant averages for each pollutant, outdoor averages measured in the city over the same time periods, averages from two other published indoor air studies, published air quality guidelines, suggested approaches to reduce the concentrations in their homes, and background information on the regulations of outdoor air pollution versus guidelines for indoor air pollutants. The results were also compared to other urban indoor air quality studies as a result of the lack of indoor air quality regulations. 

A brief one-page questionnaire was also included in the mailing, which participants were asked to complete and either bring with them to the in-person data report-back meeting, or mail back to the research team via an included post-marked envelope. The mailed questionnaire is included in Figure S1. Participants were invited to attend the in-person data report-back meeting at the GreenRoots facility, which was organized by the research team, which included GreenRoots leadership. The meeting was held on a weekday evening, and both child-care and dinner were provided for all attendees. To encourage attendance, all of the respondents were entered into a raffle for a $25 gift card to a local grocery store. All of the materials in the data report-back, evaluation, and meeting were translated into Spanish for the 26 participants (36% of participants) who were Spanish speakers. We obtained approval for this evaluation and all related report back communications from Harvard T.H. Chan School of Public Health’s Institutional Review Board via modifications approved after expedited review (CR15-1756-04).

As participants entered the report-back meeting, they were asked to sign in with a research team member and to return their completed mailed questionnaire about their personal results. Each questionnaire had a unique participant identification number, enabling us to link their report-back responses to their survey data and environmental measurements. During the meeting, the research team presented a summary of the study goals, the methods used to monitor the indoor air pollutants, and the information provided within their data report-back packets describing key pollutants and their sources within the home and in the community. Time was provided for participants to look through their personal report-back packets, to discuss with each other, and to ask the research team questions. At the end of the meeting, participants were given a second one-page questionnaire that included four questions repeated from the first questionnaire, and additional questions about their understanding of the data. Participants were asked to complete and return the questionnaires to the research team before leaving, and they were also coded with each participant ID. This questionnaire is included in Figure S2.

Before the in-person meeting, four members of the research team were trained to take notes on the engagement and participation of the meeting attendees by using ethnographic field notes methods [17,18]. These notes were collected and compiled in the week after the meeting. Additionally, at the end of the meeting, the research team provided participants with a phone number where they could leave an optional anonymous voice message with any additional feedback that they thought of after leaving the meeting, or did not want to share in writing or at the meeting. 

The framework for participant comprehension follows that outlined for document literacy by Murray et al. in the Technical Report on the First International Adult Literacy Survey. This includes an assessment of, “the knowledge and skills required to locate and use information contained in various formats, including … table, and graphics” [19]. We considered several facets of document comprehension, including locating, integrating, and generating information based on the content provided in the data report-back [20]. These three skills were considered to be successive levels of increasing comprehension of the information contained within the report-back packet. “Locating” tasks are those that “require the reader to find information in the text based on conditions or features specified in the question or directive” [20]. “Integrating” tasks require the participant to “compare or contrast two or more pieces of information”, and “generating” tasks require participants to “produce a …response by processing information from the (document) and by either making text-based inferences or drawing on their own background knowledge” [20]. All three tasks were evaluated via questions in both the mailed and post-meeting surveys.

### 2.2. Questionnaire Development

Questionnaires were developed based on the goals of the following: (1) characterizing participants’ valuation of the report-back process, (2) their understanding of the information within the report-back, and (3) their plans to take actions to reduce the air pollutants in their homes. The completion of at least one of the questionnaires was also considered an indication of engagement in the report-back process. Questions on the mailed questionnaire about valuation asked participants about whether they felt as though they understood how PM_2.5_ and NO_2_ can be harmful to health, whether they were generally concerned about air pollution in Chelsea, and whether they understood how to reduce air pollutants in their home. 

**Figure 2:**
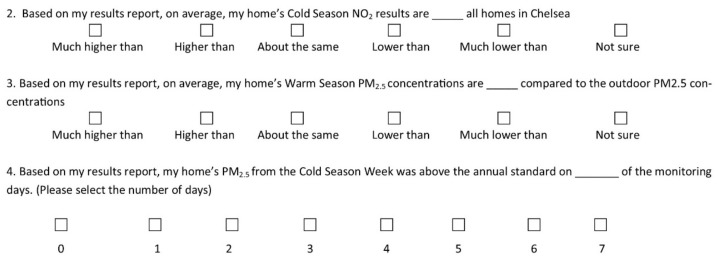
Visual of the data comprehension questions asked of the participants.

Participants were asked to identify and interpret their personal data within their report-back packet via four questions. These four questions were constructed based on the three levels of document literacy—locating, integrating, and generating—outlined above [19,20,21]. Within this framework, the ability to integrate information in the document required additional comprehension beyond locating information, and generating information required the highest level of document comprehension, incorporating the prerequisite skills of locating and integrating information. A visual of the four data comprehension questions is displayed in Figure 2.

**Figure 3:**
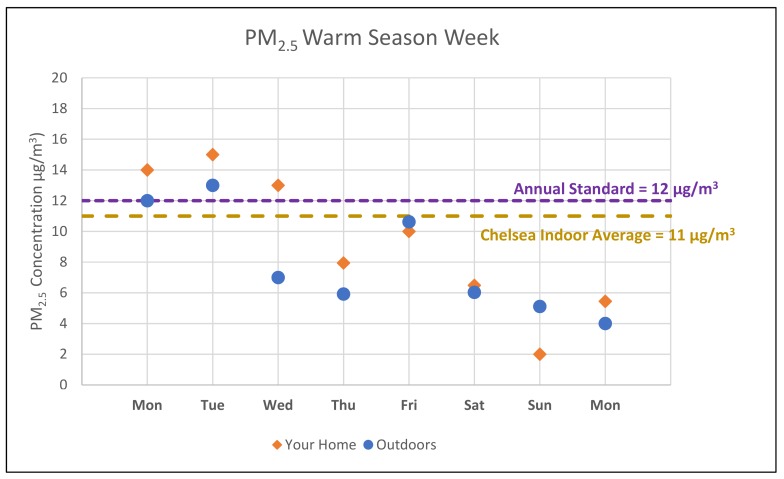
Example of the graphical component from report-back packets.

“Locating” was evaluated by asking participants to find a specific piece of information contained within their packet, namely: how their indoor concentrations of NO_2_ during the cold season related to the concentrations measured in Chelsea. “Integrating” was evaluated via a question asking participants to compare their average warm season PM_2.5_ concentrations to the outdoor concentrations measured relying on a graphical component (shown in Figure 3) of the report-back packet, and a second question asking participants to count how many days their cold season PM_2.5_ concentrations were above the EPA annual outdoor standard, also relying on a graphical component. Finally, participants were asked to “generate” information by identifying a data pattern within the packet, and noting whether they expected their NO_2_ concentrations to be higher in the summer or winter of the following year. All of the above question response options were Likert-style scales [22].

The final question on the questionnaire was open-response style, and asked participants to describe actions (if any) they planned to take to reduce concentrations of PM_2.5_ and NO_2_ in their homes.

The questionnaire completed by participants at the end of the report-back meeting included the same four data comprehension questions, as well as questions about whether participants felt as though they understand how PM_2.5_ and NO_2_ may harm their health, whether they are generally concerned with air pollution in Chelsea, and whether they understand how to reduce the pollutants in their homes. These additional questions were all five point Likert-style scales indicating level of agreement [22].

### 2.3. Engagement Analysis

Participants were categorized as either respondent or non-respondent. Anyone who returned at least one of the questionnaires, or attended the meeting, was considered a respondent participant, and categorized in our analysis as engaging with the report-back information. Tests of significance for demographic differences between the respondent and non-respondent participants were t-test or Chi-square test of independence for continuous and categorical variables, respectively [23]. Respondent and non-respondent participants were also compared for their measured indoor air concentrations of PM_2.5_ and NO_2_ for both the warm season and cold season, to determine whether those who were engaging in the data report-back process were exposed to higher or lower concentrations of the measured air pollutants. We tested the difference in 24-h averaged concentrations between the two groups using a Z-test [24].

During the meeting, there were two activities to evaluate engagement. First, participants were asked to sign in so we could track who attended (and were therefore respondents). Second, the research team members previously trained to take ethnographic field notes recorded verbal and physical indications of engagement by the participants throughout the meeting [25]. These included participants’ actions throughout the meeting, including visual signs of attention towards the presentation, body language, and performing actions suggested by the presenters (such as reviewing a specific page in their report-back packet) [26]. In an acknowledgement of the role of culture on non-verbal behavior [27], the notes on engagement were informed by insider or emic knowledge of the community held by one of the presenters from Chelsea. Her knowledge both informed the research team of the preferred style of communication for many members of this community, and confirmed the accuracy of observations recorded by the data collectors as signs of engagement.

### 2.4. Comprehension Analysis

For each of the three levels of comprehension, we sought to evaluate whether the participants’ interpretation of their data matched that of the research team. Underpinning this approach was the assumption that if the research team communicated the data effectively, participants would interpret the data similarly to the team. Three members of the research team with substantial training and experience in environmental health were separately provided blank versions of the same questionnaires completed by participants. They read through each data report-back packet (n = 72) and provided responses to the four data comprehension questions as though each household’s data were their own. For each of the four questions regarding data comprehension, consistency in response by the research team members was calculated via an intraclass correlation coefficient (ICC). The ICC measured the absolute agreement of the team (rather than consistency) and used a two-way random effects model, because all of the interpretations were provided by the same three raters [28]. The null hypothesis tested via this ICC was that there was no agreement in interpretation among the three raters. Questions with ICC values below 0.5 were considered to be poor and were removed from further analysis because of the heterogeneity of response by the research team [28].

The locating question had six response options, namely: whether their personal concentrations were “much higher than”, “higher than”, “about the same as”, “lower than”, or “much lower than” all of the measured indoor concentrations in Chelsea, or the option of “not sure”. The scale was collapsed from five levels of comparison to three, as follows: lower than, higher than, or about the same as (or not sure). This was done for both the mailed and post-meeting questionnaires. Two 3 x 3 tables were created for the mailed and post-meeting surveys. The tables depict whether each response indicated that they believed their cold season NO_2_ concentrations were “higher than”, “the same as”, or ”lower than” those reported for Chelsea overall.

The integration question had the following ten response options: The numbers 0 through 8 as a count of the number of days their home’s particulate matter concentration exceeded the annual standard, and the option of “not sure”. For each participant, the difference between their response and each research member’s response was calculated. This was done for both the mailed questionnaire and the meeting questionnaire. This created two datasets representing the differences in responses between participants and the research team members for both pre-meeting and post-meeting responses. The average difference in responses was calculated for the pre-meeting and post-meeting questionnaires, and compared via the Wilcoxon signed-rank test to determine whether the average difference in differences between the two questionnaires was zero [29].

The “generation” question had three response options of “summer”, “winter”, or “not sure”. A binary variable was generated to indicate whether the participant’s response matched each of the reviewers’ responses. The McNemar’s test was used to compare the agreement in response of the two seasons by the research team and the participants before and after the meeting [30]. A separate count of “not sure” responses was tallied.

### 2.5. Motivation to Action

We evaluated participant motivation to action to determine whether the respondent participants were inspired to incorporate and apply some information from the report back to their personal lives. This was evaluated in the following two ways: by the number of participants self-reporting plans to take action (on questionnaires and at the meeting through comments and affirmative responses), and by the relevance of the proposed actions to reducing indoor air pollutant concentrations.

On both the mailed and meeting questionnaires, the final question was an open-ended response style question in which participants were asked to list what actions, if any, they planned to take after either receiving the data report-back packet and/or attending the report-back meeting. The number of participants listing actions was counted for both questionnaires, and all of the responses compiled. The responses are listed in Figure S3. At the end of the in-person meeting, participants were verbally asked whether they were motivated to “change anything” after attending the meeting. Participants’ responses were recorded by the research team note-takers.

### 2.6. Valuation Analysis

The mailed and in-meeting questionnaires contained questions about participants’ perception of the value of the report-back process and the information presented within. Some of these questions varied slightly between the mailed questionnaire and the meeting questionnaire. All of the valuation questions had five Likert-style scale answer options, indicating degree of agreement from “strongly disagree” to “strongly agree” [22]. The percent of participants answering for the different Likert scale options were calculated for each of these questions. Additionally, the presenters facilitated an end of meeting discussion at the conclusion of the in-person report-back meeting. Participants were asked questions about their agreement with statements regarding their experience during the report-back meeting, and to indicate their agreement via hand-raising. Research team note-takers recorded the number of hands raised for each question.

### 2.7. Qualitative Analysis

Line by line analysis was done on the field notes from the meeting note-takers using open coding and grounded theory methodology [25]. Notes were organized and summarized by meeting section. No distinction was made between the research participants and any family members that they brought to the meeting for this analysis. The components from the meeting that worked well or poorly were also identified and summarized. One participant used the voicemail to leave feedback regarding the usefulness of the report-back meeting.

All of the statistical analyses were performed in RStudio software, version 1.1456. The completed questionnaires were linked to individual participants’ demographic data, as well as their baseline assessment responses and their measured indoor air pollutant concentrations.

## 3. Results

### 3.1. Engagement

Of the 72 HOME Study participants in Chelsea, one was lost in follow-up. Report-back materials were prepared for 71 participants. Of these, 31 were categorized as respondent (having engaged in at least one component of the report-back process). Of those, 28 returned the first mailed questionnaires (three in Spanish) via mail or in-person at the meeting, 16 attended the in-person meeting, and 14 returned the meeting questionnaire.

Table 1 summarizes the demographic information of the participants who engaged and did not engage in the data report back process. Respondent participants were significantly different from those who were non-respondent in terms of their age, race, ethnicity, highest degree of completed education, and language preference. No significant difference was found for the annual household income.

The respondents also had significantly higher measured 24-h average indoor concentrations of NO_2_ in the summer (but not winter), and PM_2.5_ in the winter (but not summer). The concentrations are displayed in Table 2.

Among the participants who attended the in-person report-back meeting, there were varying levels of engagement with the presentation and the interactive time. The following behaviors were documented by the research team note-takers as indications of engagement during the meeting: looking at the screen and/or watching the presenter, staying with the presenter as she presented each section of the report-back packet, complying with and following presenter instructions, responding to questions both verbally and non-verbally, commenting when asked, asking questions at different times in the presentation, asking “relevant” questions, facial expressions reacting to the content being presented, nodding of heads during the presentation, lack of side conversations, referring to and/or looking through the report-back packets during the presentation, and taking pictures of presenter slides. 

### 3.2. Understandability

Of the 71 report-backs, there were some participants for whom there was only one season of data (either warm or cold season missing). As a result, questions referring to season-specific data in the report-back evaluation questionnaires were unanswerable for some participants. A detailed breakdown of the numbers of participants responding to each question, and their responses, is provided Table S1 of the Supplementary Materials. 

Three of the four data interpretation questions had an acceptable agreement in response among research team members via ICC, as shown in Table S2 of the Supplementary Materials. The second locating question had an ICC below the cut off of 0.5, indicating poor agreement among the research team in their interpretation of the participants’ data or the question itself. This question was removed from further analysis. The three remaining questions had an acceptable agreement in response by the research team members, indicating well-formulated questions. 

Detailed accounts of responses to the remaining locating questions are provided in Table S3 of the Supplementary Materials, indicating whether participants and reviewers interpreted the data for each report back’s concentrations as “higher than”, “the same as”, or “lower than” all of the measured indoor concentrations in Chelsea. There was a 58.3% agreement among participants and research team members in the pre-meeting survey, and a 66.7% agreement in the post-meeting survey. Five participants indicated that they were unsure of the answer in the mailed survey, while none indicated that they were unsure on the post-meeting survey. 

On the mailed questionnaire, 25 participants provided responses to the integrating question, with seven indicating that they were unsure of the correct answer. The question asked participants to fill in the number of days their data were above the annual standard. Of 17 responses compared with the research team responses, the average difference in the number of days was three. On the post-meeting questionnaire, 13 participants answered the same question, with none indicating that they were unsure of how to answer. However, one participant skipped the question. The average difference in the number of days participants vs research team members perceived each participant’s cold season week to be above the annual standard was zero days after the meeting. The Wilcoxon signed rank test indicated that this shift from three days difference to zero days difference in response was statistically significant with a *p*-value of <0.001.

In the mailed questionnaire, 26 participants answered the generating question, with 10 indicating that they were unsure of the correct responses. This left sixteen participant responses to compare with the research team members. Detailed information regarding the comparisons between participant and research team members’ responses is provided in Table S4 of the Supplementary Materials. On the mailed questionnaire, 73.3% of the comparisons were matches, and on the post-meeting questionnaire, 75% were matches. Matches between the participant and the research team member indicate that the participant was able to correctly identify the pattern regarding the season in which their personal indoor NO_2_ concentrations would be higher on average in the future. This was indicative of the participants comprehending data to such a degree that they were able to not only identify information, but do project a pattern from that data into the future. Both before and after the data report-back meeting, there were more matches between participants and research team members than there were no-matches. 

### 3.3. Actionability

Thirteen participants indicated that they planned to take action before the report-back meeting. Five participants indicated that they planned to take action after attending the report-back meeting. All participant-proposed actions from the two questionnaires are listed in Figure S3. Additionally, several participants verbally indicated that they planned to take action during the end of the meeting discussion. Participants referred to changes they would make to the products they use in their homes (such as scented candles, bath soap, and detergents), or that they would increase the ventilation of their home. This was also noted by one of the meeting presenters, who commented that the participants were interpreting information gleaned from the meeting and applying it to their personal care and home products.

### 3.4. Valuation

When asked if they valued learning about how PM_2.5_ and NO_2_ can affect their health in the mailed questionnaire, one participant strongly disagreed, eight participants indicated they agreed, eighteen participants strongly agreed, and one participant was neutral among respondents. When asked if they valued that learning about the presence of the pollutants specifically in their home can affect their health, one participant strongly disagreed, 10 participants agreed, and 17 participants strongly agreed. In response to their belief in their personal understanding of how to reduce pollutants in their home before attending the meeting, four participants indicated that they were unsure, one participant strongly disagreed, two participants disagreed, ten participants agreed, and six participants strongly agreed. After the meeting, nine participants felt neutral, ten participants agreed, and five participants strongly agreed. During the end of the meeting discussion, when asked whether attendees learned something new during the in-person meeting, 10 people raised their hand in agreement. 

## 4. Discussion

This work builds upon prior evaluations of data report-back efforts by incorporating quantitative elements into a framework of formal report-back evaluation. This component provides the ability to compare the participants’ interpretation of their personal report-backs to the interpretation of the research team. Doing so allows a means of determining the efficacy of the data communication efforts in terms of whether the messages that the research team identified from the data were those that were received by participants.

### 4.1. Engagement

The demographic comparisons of respondent and non-respondent participants indicate that there were significant differences between those who engaged in the data report-back process and those who did not in terms of their race, ethnicity, age, language preference, and educational attainment. Specifically, respondents were generally older, tended to prefer English, had higher levels of educational attainment, did not identify as Hispanic or Latin-x, and were white. They also tended to have lower measured pollutant concentrations in their homes. Although efforts were made to ensure that the meeting was accessible for all participants, through multiple pre-meeting communications and the provision of food and childcare on site, it is clear that there may still have been barriers to participation. It is necessary to ensure that we are providing data in a way that is understood by vulnerable populations. Future efforts should include approaches to identify and address barriers to engagement so as to ensure that vulnerable populations (e.g., higher concentrations) are reached. 

### 4.2. Understandability 

The inconsistency in the interpretation of one of the questions indicated a poorly worded question. Our pilot of the questionnaire was not sensitive enough to determine the clarity of individual questions, and rather indicated that overall the questions were clear. There was an overall reduction in participants’ responding that they were unsure after attending the meeting—which could indicate information gain over the course of the meeting. It could alternatively indicate that participants who experienced less difficulty with the materials are those who answered the post-meeting evaluation. Additionally, there was a small sample size, and smaller overlap between the two groups who answered both questionnaires. The reasoning behind the increase in answers matching the responses of the research team is also unclear because of these reasons. However, the general increase in matches indicate that there was some information gained by participants over the course of the meeting. This was further substantiated by comments made by participants over the course of the meeting, in which they identified specific exposure reduction actions that they could take in their own homes. 

### 4.3. Valuation

Overall, the respondents indicated that they agreed or strongly agreed that they valued learning about the pollutants in their home, and how they may impact their health. It would have been valuable to collect information from the participants who did not engage in the data report-back process to determine whether lower perceived value of the report-back process prevented them from engaging. A potential approach to determining the valuation of the data report-back of all of the participants would be to collect that information at the time of baseline data collection at the beginning of the study so as to provide responses for all at the start. 

### 4.4. Actionabiltiy

Overall, the actions that participants reported that they planned to take were relevant to the information communicated through the report-back process. Additionally, the questions asked during the meeting indicated that participants were seeking clarifying information so as to guide their approaches to reduce exposures within their own homes. This evaluation was limited to characterizing participants’ intention to reduce exposures. It would be useful in future report-back evaluation efforts to include a follow-up component to assess whether participants later took action. 

## 5. Conclusions

To our knowledge, this is the first mixed-methods evaluation of an exposure assessment report-back process. The combination of qualitative and quantitative information allowed for a cross-validation of findings. The qualitative information collected during the in-person meeting was particularly useful in verifying the indication from the surveys that respondents were generally interpreting their data correctly. These qualitative data were particularly useful in supplementing the results of the quantitative questionnaire data, which in some cases were not sufficient to clearly indicate participant information gain because of limited pre- and post-paired responses. 

However, there were substantial demographic differences between those who engaged in the report-back process as compared to those who did not—indicating room for improvement in future report-back efforts. Respondents were not representative of the sample population or the overall demographics of Chelsea, as indicated by the lack of Spanish-speaking participants at the meeting, and under the representation of non-white and Hispanic or Latin-x populations. This difference of representativeness may represent bias in the outreach and materials that prevented all participants from engaging equally. Furthermore, those who did not engage had significantly higher indoor air pollutants than those who did engage. Increasing outreach to participants with higher concentrations can be a useful first step in addressing indoor air quality disparities by ensuring that vulnerable populations are included.

The findings from this process evaluation provide additional insight for improvements in future efforts. Specifically, carefully designed pilot-testing evaluation materials with members of the intended audience can provide awareness of the weaknesses in the materials, and an appropriateness of the outreach and evaluation components.

## Figures and Tables

**Table 1:** Demographics of the respondents and non-respondents to the report-back process.

	Non-Respondent	Respondent	*p*-value
	n = 41	n = 31	
**Educational Attainment**			
			<0.001
Less than high school diploma or GED	15 (36.6)	3 (9.7)	
High school diploma or GED	8 (19.5)	3 (9.7)	
Some college but no degree	5 (12.2)	5 (16.1)	
Associate degree	7 (17.1)	1 (3.2)	
Bachelor’s degree	5 (12.2)	8 (25.8)	
Post graduate degree (masters or doctoral)	1 (2.4)	11 (35.5)	
**Hispanic or Latin-x**			
No, not Hispanic or Latin-x	12 (29.3)	25 (80.6)	<0.001
**Race**			
			<0.001
American Indian or Alaska Native, Black or African American, Native Hawaiian or Other Pacific Islander, Other	0 (0.0)	1 (3.2)	
Asian	1 (2.4)	1 (3.2)	
Black or African American	1 (2.4)	3 (9.7)	
White	9 (22.0)	21 (67.7)	
Other	30 (73.2)	5 (16.1)	
**Participant Language Preference: Spanish**			
Spanish	19 (46.3)	3 (9.7)	0.002
**Annual Household Income**			
			0.098
Less than $10,000	11 (26.8)	4 (12.9)	
$10,000–$24,999	11 (26.8)	3 (9.7)	
$25,000–$49,999	4 (9.8)	6 (19.4)	
$50,000–$99,999	7 (17.1)	9 (29.0)	
$100,000+	3 (7.3)	7 (22.6)	
Refused to answer	4 (9.8)	1 (3.2)	
Do not know	1 (2.4)	1 (3.2)	
**Average Age of Participant**			
Mean (SD)			0.007
	47.63 (14.51)	57.34 (14.41)	

**Table 2:** Average weekly PM_2.5_ and NO_2_ concentrations by season for the respondents and non-respondents.

**PM_2.5_ (ug/m^3^)**			
	**Respondents**	**Non-Respondents**	***p*-value**
**Summer**	4.6	5.7	0.1683
**Winter**	3.4	5.9	<0.001
**NO_2_ (parts per billion )**			
	**Respondents**	**Non-Respondents**	***p*-value**
**Summer**	10.5	20.8	<0.001
**Winter**	30.9	28.2	0.3392

## Supplementary Materials

The following are available online at https://www.mdpi.com/1660-4601/16/21/4183/s1, Figure S1: Data Report-Back Evaluation Mailed Questionnaire. Figure S2: Data Report-Back Evaluation Meeting Questionnaire. Figure S3: Actions Participants Reported They Planned to Take Before and After Report-Back Meeting. Table S1: Number of responses to data interpretation questions by research team members and participants; Table S2: Intraclass correlation coefficients for research team responses to data comprehension questions; Table S3: Participants’ and research team members’ responses to ‘locating’ question; Table S4: Matching of participants’ and research team members’ responses to ‘generating’ information.
