# Supplementary material for: A Mixed Methods Evaluation of Sharing Air Pollution Results with Study Participants via Report-Back Communication
Source: Int J Environ Res Public Health. 2019 Oct 29;16(21):4183. doi: 10.3390/ijerph16214183 (PMC6862165; doi:10.3390/ijerph16214183)
Supplement: Supplementary file 1 [file ijerph-16-04183-s001.pdf]

Supplementary Materials

Figure S1: Data Report-Back Evaluation Mailed Questionnaire.

Participant ID#: _____________

CRESSH HOME

Results Questionnaire

Please read through your study results and then answer the questions below by selecting the single answer you believe is most accurate, or writing in your answer in the space provided. Bring your completed questionnaire to the meeting on **August 13th** for a chance to be entered into a raffle for a $25 gift card to Market Basket.

1. I value learning about how particulate matter ($PM_{2.5}$) and nitrogen dioxide (NO_2) can affect my health.

☐ Strongly agree ☐ Agree ☐ Neutral ☐ Disagree ☐ Strongly disagree

2. I value learning about how the presence of $PM_{2.5}$ and NO_2 *in my home* may affect my health.

☐ Strongly agree ☐ Agree ☐ Neutral ☐ Disagree ☐ Strongly disagree

3. Based on my results report, on average, my home's Cold Season NO_2 results are _____ all homes studied in Chelsea.

☐ Much higher than ☐ Higher than ☐ About the same ☐ Lower than ☐ Much lower than ☐ Not sure

4. Based on my results report, on average, my home's Warm Season $PM_{2.5}$ concentrations are _____ compared to the outdoor PM2.5 concentrations measured in Chelsea.

☐ Much higher than ☐ Higher than ☐ About the same ☐ Lower than ☐ Much lower than ☐ Not sure

5. Based on my results report, my home's $PM_{2.5}$ from the Cold Season Week was above the annual standard on _______ of the monitor-ing days. (Please select the number of days)

☐ 0 ☐ 1 ☐ 2 ☐ 3 ☐ 4 ☐ 5 ☐ 6 ☐ 7

6. Based on the NO_2 results presented on page 5, I would expect NO_2 concentrations next year to be higher in my home, on average, in:

☐ Summer ☐ Winter ☐ Not Sure

7. I understand how to reduce the pollutants in the air in my home

☐ Strongly agree ☐ Agree ☐ Neutral ☐ Disagree ☐ Strongly disagree

8. Since living in Chelsea, I have spent time trying to learn about air quality in Chelsea (for example, searching online, reading a book or brochure, attending a meeting or presentation).

☐ Strongly agree ☐ Agree ☐ Neutral ☐ Disagree ☐ Strongly disagree

9. What, if any, actions might you take after reading this report? Please explain your response.

Figure S2: Data Report-Back Evaluation Meeting Questionnaire.

Participant ID#: ___________

CRESSH HOME

Meeting Questionnaire

Please complete these questions when prompted to do so during this evening's meeting, and return to presenters at the end of meeting before you leave.

1. I understand how PM_{25} and NO_2 may be harmful to health.

☐ Strongly agree ☐ Agree ☐ Neutral ☐ Disagree ☐ Strongly disagree

2. Based on my results report, on average, my home's Cold Season NO_2 results are _____ all homes in Chelsea

☐ Much higher than ☐ Higher than ☐ About the same ☐ Lower than ☐ Much lower than ☐ Not sure

3. Based on my results report, on average, my home's Warm Season PM_{25} concentrations are _____ compared to the outdoor PM2.5 concentrations

☐ Much higher than ☐ Higher than ☐ About the same ☐ Lower than ☐ Much lower than ☐ Not sure

4. Based on my results report, my home's PM_{25} from the Cold Season Week was above the annual standard on _______ of the monitoring days. (Please select the number of days)

☐ 0 ☐ 1 ☐ 2 ☐ 3 ☐ 4 ☐ 5 ☐ 6 ☐ 7

5. Based on the NO_2 results presented on page 5, I would expect NO_2 concentrations next year to be higher indoors in my home, on average, in:

☐ Summer ☐ Winter ☐ Not Sure

6. After reviewing my results during this meeting, I feel confident in my understanding of my home's air quality results after receiving the report in the mail

☐ Strongly agree ☐ Agree ☐ Neutral ☐ Disagree ☐ Strongly disagree

7. How concerned are you with air pollution in Chelsea?

☐ Very Concerned ☐ Concerned ☐ Not Concerned

8. I understand how to reduce the pollutants in the air in my home

☐ Strongly agree ☐ Agree ☐ Neutral ☐ Disagree ☐ Strongly disagree ☐ Not sure

9. What, if any, actions might you take after attending this meeting? Please explain your response.

Figure S3: Actions Participants Reported They Planned to Take Before and After Report-Back Meeting.

**Actions Listed Before Attending Report-back Meeting**
"Clean screening before opening windows. Only possible source I can think of for summer particular pollutants"
"Find out ways to reduce pollen in the house. Be more environmental educated. Do more research"
"Check furnace to see if could make more efficient - cleaned more often (oil) in cold season. In warm season - don't know"
"Research online/read up on the growing Chelsea population"
"Do more online research, and also talk to the study organizer/leads to understand how I can reduce air pollution in my home. Thank you for this opportunity to participate."
"Replaced air purifier filters, changed HVAC filter. Decided not to burn candles and keep fan on after using stove. I'm still looking to understand why my winter NO_2 values were so high. Thank you for raising awareness!! :)"
"I will be much more aware of pollutants and how to reduce and/or avoid them"
"Hope to get ideas about how to reduce indoor air pollution; not sure at this point, I need to inform myself"
"Stop buying candles. Limit open windows in summer: more careful cooking and burning food"
"I wish there were more participants and that businesses near me participated (gas company, hide and fur co, etc.)
"Continue my current efforts. Maybe tackle the dump! In Chelsea why is that allowed to be in the middle of a residential area!!"
"Avoid use of candles and air fresheners. Open windows and use the stove fan."
"Use vent when cooking with gas; stop burning candles and incenses - stop smoking; I would like to know how to keep the outside as much as I can clean and stop pollution. Thank you for the results."
**Actions Listed After Attending Report-back Meeting**
"Clean vents/ window screens/fans/dryer vents more often"
"1. Try to improve boiler's air intake - How? From just pulling air in from the air in the room to having a more "fresh air" intake. 2. Explore changing heating system - oil -> clean air (electricity, etc.)."
"I use the wick like air fresheners. I must think of another alternative to keep my air in my home refreshed"
"Increase ventilation during cold season to lower NO_2 levels"
"Love the visual of the m^3. Helpful for understanding this concept. Everywhere I shop I see candles, Febreze, and other air fresheners and I realize it is important to better understand how these impact air quality, and this presentation has been very helpful in increasing my understanding. I would like to

educate myself further about green cleaning products and this presentation was a good introduction. This is an area rich for further development"

Table S1: Number of responses to data interpretation questions by research team members and participants.

Question Task	Number of Participants with Seasonal Data Missing	Number of Participant Responses on Mailed Questionnaires (*number of participants responding 'not sure'*)	Number of Participant Responses on Post-Meeting Questionnaires (*number of participants responding 'not sure'*)	Number of Report-backs Interpreted by Research Team Members
**Locating (1)**	**Text:** "Based on my results report, my home's Cold Season NO_2 results are ___ all homes studied in Chelsea"			
	5	25 (*5*)	14 (*1*)	66
**Locating (2)**	**Text:** "Based on my results report, my home's Warm Season $PM_{2.5}$ concentrations are __ compared to the outdoor $PM_{2.5}$ concentrations measured in Chelsea"			
	10	27 (*6*)	14 (*0*)	61
**Integrating**	**Text:** "Based on my results report, my home's $PM_{2.5}$ from the Cold Season Week was above the annual standard on __ of the monitoring days."			
	5	25 (*7*)	13 (*0*)	66
**Generating**	**Text:** "Based on the NO_2 results presented on page 5, I would expect NO_2 concentrations next year to be higher in my home, on average, in: "			
	12	26 (*10*)	14 (*1*)	59

Table S2: Intraclass correlation coefficients for research team responses to data comprehension questions.

Question Task	Number of Reports Interpreted by Research Team	ICC	P-value	95% CI
**Locating (1)**	**Text:** "Based on my results report, my home's Cold Season NO_2 results are ___ all homes studied in Chelsea"			
	66	0.886	<0.001	0.834<ICC<0.925
**Locating (2)**	**Text:** "Based on my results report, my home's Warm Season $PM_{2.5}$ concentrations are __ compared to the outdoor $PM_{2.5}$ concentrations measured in Chelsea"			
	61	0.366	<0.001	0.201<ICC<0.528
**Integrating**	**Text:** "Based on my results report, my home's $PM_{2.5}$ from the Cold Season Week was above the annual standard on __ of the monitoring days."			
	66	0.924	<0.001	0.889<ICC<0.950

**Generating**	**Text: "**Based on the NO2 results presented on page 5, I would expect NO2 concentrations next year to be higher in my home, on average, in: "			
	59	0.712	<0.001	0.597<ICC<0.805

Table S3: Participants' and research team members' responses to 'locating' question.

**Mailed Questionnaire**				
		**Reviewers**		
		**Lower**	**Same**	**Higher**
**Participants**	**Lower**	23	2	5
	**Same**	7	5	3
	**Higher**	3	5	7

**Post-Meeting Questionnaire**				
		**Reviewers**		
		**Lower**	**Same**	**Higher**
**Participants**	**Lower**	20	4	3
	**Same**	0	0	3
	**Higher**	0	3	6

Table S4: Matching of participants' and research team members' responses to 'generating' information question.

**Mailed Questionnaires**			**Post-Meeting Questionnaires**		
**Match**	**No Match**	**Unsure**	**Match**	**No Match**	**Unsure**
33	15	10	27	9	1
